# Cross-regulatory interaction between the HPI axis and appetite regulation in Atlantic salmon (*Salmo salar*) parr under chronic and acute stress

**DOI:** 10.3389/fendo.2025.1685870

**Published:** 2025-11-05

**Authors:** Floriana Lai, Ivar Rønnestad, Lars Helge Stien, Angelico Madaro

**Affiliations:** ^1^ Department of Biological Sciences, University of Bergen, Bergen, Norway; ^2^ Institute of Marine Research, Matredal, Norway

**Keywords:** appetite, hypothalamus, unpredictable chronic stress, acute stress, melanocortin system, HPI axis

## Abstract

Reduced feed intake is a common stress response in Atlantic salmon (*Salmo salar*), yet the interaction between the stress-related hypothalamic–pituitary–interrenal (HPI) axis and the appetite-regulating hypothalamic melanocortin system remains poorly understood. This study explored the potential cross-regulatory interaction between these two systems by assessing feed intake, gastrointestinal content, and hypothalamic mRNA expression of *crf1* paralogs, a key factor in stress handling, and appetite-related genes *npy*, *agrp1*, *pomc*, and *cart* in Atlantic salmon parr exposed to 21 days of unpredictable chronic stress, followed by a novel acute stressor. Our results indicated that regulation of the HPI axis and appetite-regulatory mechanisms appeared to depend on the type and duration of stress. Chronic stress reduced feed intake and gut content and increased feed conversion ratio, without changes in hypothalamic *crf1* paralog expression but with reduced orexigenic *npya1* transcript levels. Simultaneously, levels of the anorexigenic *cart2b* transcript variant were also reduced, possibly reflecting a compensatory response to prolonged appetite suppression. In contrast, exposure to the novel acute stressor induced a transient increase in *crf1* paralogs and upregulated orexigenic neuropeptides *npya1* and *npya2*, suggesting compensatory regulation to counteract stress-induced anorexia. In contrast, *cart2b* expression normalized to control levels, possibly reflecting an acute stress-induced compensatory response restoring appetite regulation. These results highlight a dynamic, stress-duration-dependent interaction between stress and appetite-regulatory systems in Atlantic salmon.

## Introduction

In Atlantic salmon (*Salmo salar*), reduced feed intake is a well-recognized indicator of stress, particularly during periods of allostatic adjustment when fish are exposed to prolonged or repeated challenges (1–5). The hypothalamic–pituitary–interrenal (HPI) axis plays a central role in coordinating the physiological stress responses in teleosts. The corticotropin-releasing factor (hereafter Crf) (6, 7) initiates a hormonal cascade that leads to cortisol production by the interrenal gland (8, 9). This response mobilizes energy reserves to help the animal cope with stress, but often comes at the cost of reduced feed intake and growth performance (8, 10, 11). Appetite regulation in teleost involves a complex interplay between hypothalamic orexigenic (e.g., *npy* and *agrp*) and anorexigenic (e.g., *pomc* and *cart*) neuropeptides, which act through the melanocortin system to modulate feeding behavior and energy homeostasis (12, 13). While both the HPI axis and the melanocortin system play critical roles in delivering the required energy to meet the metabolism demands, the extent and mechanisms of their interaction remain largely unexplored.

The involvement of the HPI axis in feed intake and appetite regulation has been shown to vary across teleost species and to be dependent on the type of stress (14, 15). In goldfish (*Carassius auratus*), a moderate increase in plasma cortisol stimulated feeding behavior, accompanied by upregulation of *npy* and downregulation of *crf1b* expression in the preoptic area (POA). In contrast, higher cortisol doses suppressed *crf1b* without affecting *npy* or feeding behavior (16). In zebrafish (*Danio rerio*) exposed to acute stress, cortisol elevation increased the expression of both orexigenic and anorexigenic neuropeptides in the whole brain (17). In rainbow trout (*Oncorhynchus mykiss*), subordinate individuals exhibited higher expression levels of *crf1* and *npy* in the POA compared to dominant fish (18). However, the absence of feed intake data prevents evaluation of whether the increased expression of these two genes is linked to appetite regulation. Chronic stress in Atlantic salmon reduced feed intake and increased *npya1* expression in the diencephalon, while *crf1* paralogs remained unchanged (19).

It has also been suggested that Crf nuclei in appetite-regulating centers in the hypothalamus, such as nucleus lateralis tuberis (NLT) and the nucleus recessus lateralis (NRL), play a key role in reducing appetite during stressful events (14). Despite their potential importance, the functional interaction between stress-responsive pathways and appetite-regulatory circuits within these nuclei remain poorly characterized. Moreover, the recent availability of data from the salmonid-specific fourth whole-genome duplication has allowed the identification of novel peptide paralogs, sparking renewed interest in their roles in appetite and stress regulation and providing new insights into the molecular mechanisms underlying feed intake regulation under stress in Atlantic salmon (20–23).

**Figure 1:**
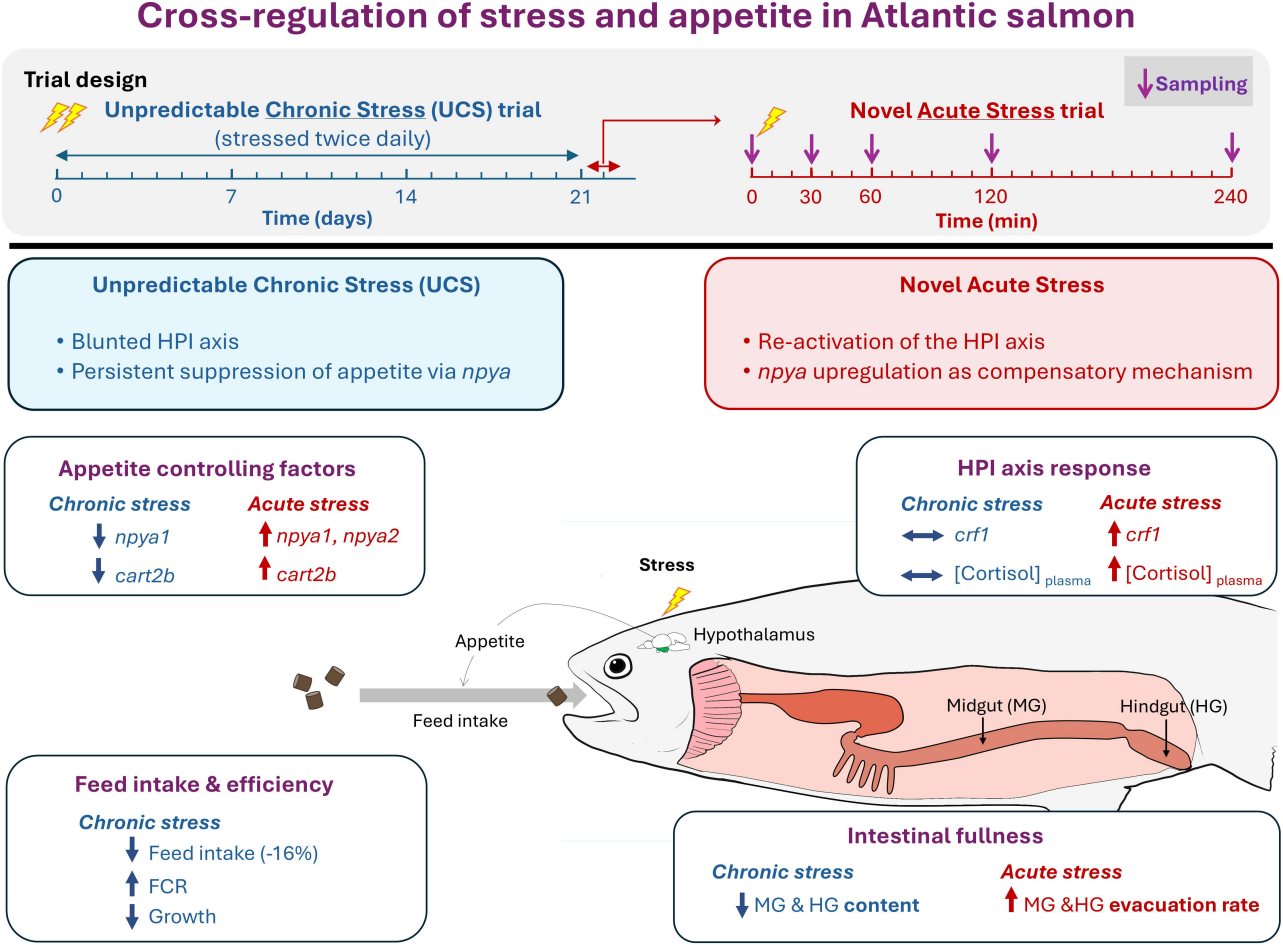
Experimental design and schematic representation of the cross-regulation between stress and appetite in Atlantic salmon (*Salmo salar*). The diagram presents the experimental timeline and illustrates the cross-regulation between the hypothalamus–pituitary–interrenal (HPI) axis activity and appetite regulation under chronic and acute stress conditions. Key findings are highlighted, including changes in hypothalamic appetite-regulating factors, HPI axis responses, feed intake and efficiency, and intestinal fullness and transit. Symbols: “↑” and “+” indicate increase; “↓” and “−” indicate decrease; “⟷” indicates no change.

In this study, we extend the findings of a recent experiment by Madaro et al. (24), which assessed performance and stress-related physiological responses in Atlantic salmon parr subjected to 21 days of chronic stress, followed by exposure to a novel acute stressor the next day. Chronic stress significantly impaired growth performance, and the HPI axis showed signs of habituation or exhaustion, likely due to repeated activation over the prolonged stress period. The acute stressor triggered a transient spike in plasma cortisol, but—likely due to prior chronic exposure—cortisol levels returned to baseline more rapidly, suggesting that the HPI axis was less reactive or more tightly regulated in response to new challenges. Based on these observations, we hypothesize that the interaction between the HPI axis and appetite-regulatory mechanisms may differ between chronic and acute stress conditions in Atlantic salmon. To test this hypothesis, we assessed the impact of stress on feed intake and gastrointestinal content at the end of the chronic stress period, alongside hypothalamic mRNA expression of *crf1* paralogs and key appetite-related genes *npy*, *agrp1*, *pomc*, and *cart* in response to both chronic and novel acute stressors. The principal findings are synthesized in **Figure 1**, while detailed descriptions of all evaluated parameters are provided in the Results section and **Supplementary Materials**.

## Material and methods

### Ethics statement

The experiment conformed to the Norwegian Regulation on Animal Experimentation and was approved by the National Animal Research Authority in Norway (FOTS ID 28400).

### Experimental design

Details of the experimental design can be found in Madaro et al. (24). In brief, 360 diploid parr of approximately 155 g were randomly allocated into six square tanks of 1 m² (400 L), with 60 fish each tank at the Institute of Marine Research Station in Matre (Norway). Fish tanks were supplied with freshwater at 12°C at a flow rate of 15 L/min, ensuring over 90% O_2_ saturation. Fish were fed a 2-mm commercial pellet (Skretting Nutra Olympic) during the first 2-week acclimation period and then increased to 3 mm for the same feed for the duration of the study, where 200 g/day were provided the initial 7 days, followed by 250 g/day until the experiment’s conclusion. The feed was provided twice a day: 60% between 9:00 and 11:00 a.m. and 40% between 1:00 and 3:00 p.m. Uneaten feed was collected to quantify intake.

### Unpredictable chronic stress

After the acclimation period, three tanks were randomly designated as unpredictable chronic stress (UCS) and daily subjected to two of seven stressors for 21 days. The stressors included the following: (1) chasing (5 min); (2) netting (5 min); (3–4) temperature shock (12–6–12°C and/or 12–19–12°C; approximately 90 min each); (5) a combination of noise, darkness, and flashlight (5 min); (6) emptying the tank (5 min); and (7) brief hypoxia (reaching ~60 O_2_% for 5 min). The remaining three tanks served as control and were left undisturbed.

### Novel acute stress and sampling protocol

At the conclusion of the UCS trial, feeders were stopped for 24 h before sampling. The following day, 10 fish from each experimental group were collected as baseline (0 min) and euthanized with an overdose of 300 mg/L Finquel^®^ vet (ScanAqua AS, Årnes, Norway), buffered with 300 mg/L sodium bicarbonate. From each group, the remaining fish were subjected to a novel acute stress test involving crowding within the tank, netting, subsequent transfer to a new single transport 100-L tank, and redistribution into eight new tanks (one for each sampling time point). After redistribution, 10 fish were sampled and euthanized at 30, 60, 120, and 240 min post-stress.

Body weight and fork length were measured, and blood was collected using 2-mL pre-heparinized syringes fitted with a 23 G needle. Blood plasma was separated by centrifugation at 13,000 rpm for 3 min and immediately cooled on ice. The fish whole brain was collected and transferred into tubes containing RNAlater (Invitrogen, Carlsbad, CA, USA). Brains were refrigerated for 24 h and stored at −80°C until RNA isolation was performed. Each fish was then meticulously dissected, and the gastrointestinal tract was divided into stomach, midgut, and hindgut using surgical clamps to prevent loss or transfer of content between the compartments as previously described by Del Vecchio et al. (25). Each segment was emptied of its internal content (feed and digesta) by gently transferring the material into pre-weighed, labeled bags (120 × 170 × 0.05 mm, VWR International, Oslo, Norway). The wet weight of the contents in each segment was measured immediately after sampling, whereas the dry weight was determined following a 48-h incubation in a dehydrator (Excalibur 10, Excalibur, USA) at 74°C to guarantee adequate dehydration.

### Feed intake and gastrointestinal content

Uneaten food was collected from each tank after 15 min post-feeding and left to dry in a colander for 10 min prior to weight measurement. Thereafter, feed intake was calculated according to Helland et al. (26) using the following formula:


$$Feed\;eaten\;\left(g\right)=\frac{\left(A\times Adm\;/\;100\;\right)-\left(W\times Wdm\;/\;R\;\right)}{Adm/100}$$

where *A* is the amount of feed provided (g), *Adm* is the dry matter content of the feed (%), *W* is the amount of feed waste collected (g), *Wdm* is the dry matter of the feed waste (%), and *R* is the recovery of dry matter of the feed waste (%).

The feed conversion rate (*FCR*) at the conclusion of the trial was calculated using the subsequent equation:


$$FCR=\frac{Feed\;intake}{W_{f}-W_{i}}$$

where *W_f_* − *W_i_* is the fish body weight (g) increment from day 0 to day 21.

The dry content from each GIT section was quantified to determine the degree of the *GIT fullness* in relation to the experimental condition:


$$GIT\;section\;fullness\;\left(\%\right)=\;\frac{STdw\;\text{or}\;MGdw\;\text{or}\;HGdw\;}{W\;-\;\left(STww\;+\;MGww+HGww\right)}*100$$

where *STdw*, *MGdw*, and *HGdw* are the dry weight (g) of the stomach, midgut, and hindgut content, respectively. *W* is the fish body weight (g), and *STww*, *MGww*, and *HGww* are the wet weight (g) of the stomach, midgut, and hindgut content, respectively.

### RNA isolation and cDNA synthesis

All brain samples from each experimental group and sampling point were carefully dissected, and the hypothalamus was collected as described in **Supplementary Figure 1**. Total RNA was extracted using TRI Reagent (Sigma-Aldrich, MO, USA), and RNA concentration and purity were measured using a NanoDrop One spectrophotometer (Thermo Fisher Scientific, Massachusetts, USA). Next, RNA quality and integrity were assessed by a 2100 BioAnalyser with an RNA 6000 Nano Lab Chip kit (Agilent Technologies, CA, USA). To remove any residual genomic DNA, 10 μg of total RNA was processed using the TURBO DNase-free Kit (Ambion Applied Biosystems, CA, USA). First-strand cDNA was synthesized from 1 μg of total RNA using SuperScript III Reverse Transcriptase (Invitrogen, CA, USA) and Oligo(dT)20 (50 μM) primers in a total reaction volume of 20 μL. The protocols were executed in accordance with the manufacturer’s guidelines.

### Real-time PCR (qPCR)

Specific primers were used for Atlantic salmon *crf1a1*, *a2*, *b1*, and *b2* (22); *npya1* and *a2* (23); *agrp1*, *pomca1*, and *a2* (27); and *cart2b* (27) (**Supplementary Table 1**). Quantification cycle (*C*_q_), primer efficiency (*E*), and melting peaks were analyzed to detect potential nonspecific products and/or primer dimers for each pair. The efficiency of the primer was assessed utilizing a 10-fold dilution standard curve (ranging from 1.00E + 07 to 1.00E + 02 copies of amplicon/μL) based on the target gene cloned onto a pCR4-TOPO vector (Thermo Fisher Scientific).

qPCRs were performed using a CFX96 Real-Time System (Bio-Rad Laboratories, CA, USA) in connection to CFX Manager Software version 3.1 (Bio-Rad, Laboratories, CA, USA). For every gene, the qPCR reaction included 10 μL of SYBR Green I Master Mix (Roche Diagnostic, Basel, Switzerland), 0.6 μL each of the forward and reverse primers (10 mM), 6.8 μL of Ultra-Pure Water (Biochrom, Berlin, Germany), and 2 μL of cDNA template (for *npya1* and *npya2*, 12 ng/reaction; for *agrp1*, *pomca1*, *a2*, and *cart2b*, 80 ng/reaction). All reactions were run in duplicate into 96-well plates (Bio-Rad Laboratories, CA). All plates included two negative controls: no-template and no-reverse transcriptase, as well as one positive control (between-plate control). The following qPCR protocol was performed: (1) 95°C for 30 s, (2) 95°C for 5 s, (3) 60°C for 25 s, and (4) repeating steps 2 and 3 for 39 times. Nonspecific products and/or primer dimers were checked by running a final melting curve analysis throughout a temperature range of 65–95°C, with increments of 0.5°C every 2 s. Subsequently, the copy number for each target gene was determined based on the respective standard curve slope and intercept using the following equation:


$$\text{Copy number}=10^{\left(\frac{Cq\;-\;intercept}{slope}\right)}$$

The copy number was normalized to the total nanograms of RNA used for each target gene.

### Statistical analysis

All statistical analyses were conducted using GraphPad Prism (GraphPad Software, version 10.4.2, San Diego, USA). Data were assessed for skewness and normality using the D’Agostino–Pearson omnibus test and Shapiro–Wilk’s tests. Body weight, length, *K*, SGR, total feed intake, and *FCR* were analyzed using a parametric *t*-test, while comparison between groups for GIT fullness and gene expression data was conducted using two-way analysis of variance (ANOVA) with treatment (control or UCS) and GIT section or sampling times as explanatory variables, and their interaction. Šídák’s multiple comparisons test was used as a post-test when GIT sections were employed as an explanatory variable, while Dunnett’s multiple comparisons test was used as a *post-hoc* test when sampling time was used as an explanatory variable. Correlation analysis between wet and dry matter content in the gastrointestinal tract was conducted using Pearson *r* correlation. For all tests, *p* < 0.05 was considered significant (*p < 0.05; **p < 0.01; ***p < 0.001; ****p < 0.0001). All data are presented as mean ± SEM unless otherwise stated. More details of the data analysis and notation can be found in the **Supplementary Materials**.

## Results

### Chronic stress effects

During the 21-day study, chronic stress led to a consistent and significant reduction in daily feed intake compared to the control group, with the UCS group consuming 16% less feed by the end of the experiment (**Supplementary Figures 2**, **3**). This reduction was reflected in a significantly higher *FCR* of 1.4 in the UCS group in contrast to the 0.6 of the control group (**Supplementary Figure 3**).

The impact of chronic stress on the feed intake of the UCS groups was also observable as differences in gastrointestinal fullness. A significant interaction between stress treatment and gut fullness sections was observed (**Supplementary Figure 4**). The UCS and control groups had comparable amounts of feed residuals in the stomach following the 24-h fasting prior to sampling, with no significant difference between the two experimental groups, while both the wet and dry contents measured in the midgut and hindgut were significantly lower in the UCS group than in the control group (*post-hoc* test, midgut wet content *p* = 0.0160; midgut dry content *p* < 0.001; hindgut *p* < 0.0001 for both wet and dry contents) (**Supplementary Figure 4**). Further analysis using Pearson’s correlation revealed a significant positive linear relationship between the wet and dry content in both experimental groups (**Supplementary Figure 5**).

There were no observable differences in the mRNA levels of the *crf1* paralogs between the control group and the UCS group after the 21-day chronic period (**Supplementary Figure 6**). However, the UCS group showed lower mRNA levels of *npya1* (*post-hoc* test, *p* = 0.0496) and *cart2b* (*post-hoc* test, *p* = 0.0217) in comparison to the control group at the 0-min sampling point (**Supplementary Figure 7**). No differences in expression were observed for *npya1*, *agrp1*, and *pomca1*.

### Acute stress effects

Acute stress exposure revealed differences in gut evacuation dynamics (**Supplementary Figure 8**). After the novel acute stressor, the UCS group showed a more pronounced reduction in midgut content at 60 and 120 min post-stress. No temporal changes were observed in the same section in the control group, or in the hindgut of either experimental group.

Exposure to the novel acute stress led to an increase in the four *crf1* paralogs, where a higher differential expression was observed in the UCS group at 120 min after stress for *crf1a1* (*post-hoc* test, *p* = 0.0307) and *crf1a2* (*post-hoc* test *p* = 0.0398), and at 240 min after stress for *crf1a1* (*post-hoc* test, *p* = 0.0257), *crf1a2* (*post-hoc* test *p* = 0.0035), *crf1b1* (*post-hoc* test *p* < 0.0001), and *crf1b2* (*post-hoc* test *p* = 0.0006) compared to the control (**Supplementary Figure 6**). Acute stress also resulted in an increase of *npya1* and *npya2* levels, which, at 240 min after the novel acute stress, were statistically significantly higher compared to the control group (*post-hoc* test, *p* = 0.0002 for *npya1* and *p* = 0.0009 for *npya2*). An increase in the *cart2b* mRNA levels was also observed in the UCS group, reaching comparable levels to the control group from 60 min after stress (*post-hoc* test, *p* = ns) (**Supplementary Figure 7**). Finally, it was shown that the expression of *agrp1*, *pomca1*, and *pomca2* was not affected by the novel acute stressors.

## Discussion

### Chronic stress effects

Chronic stress in fish is known to disrupt physiological homeostasis, particularly through dysregulation of the HPI axis and appetite-related pathways (14, 15). The purpose of exposing fish to multiple stressors in a random and unpredictable manner used in this study was to prevent habituation. This approach enables the investigation of the mechanisms that drive and regulate the HPI axis physiology and behavior under chronic stress conditions. In such circumstances, a failure to attenuate the mediators of the stress response may lead to allostatic overload, resulting in the “wear and tear” of the body (28, 29). Our findings demonstrate that the UCS protocol effectively prevented habituation to the stressors, providing new evidence of cross-regulatory interaction between the HPI axis and appetite regulation in Atlantic salmon.

Chronic exposure to unpredictable stressors led to a marked reduction in daily feed intake, consistent with previous findings in salmonids. In brown trout (*Salmo trutta*), exposure to a novel environment reduced the feed consumption (30), and rainbow trout subjected to daily brief handling showed suppressed appetite (31). Similarly, in Atlantic salmon, repeated stress exposure suppressed appetite, likely through neuroendocrine alterations involving the HPI axis (3, 32). Beyond handling and social stress, chronic exposure to suboptimal oxygen and temperature further impaired appetite in Atlantic salmon, likely via metabolic stress and hormonal signaling pathways (4, 5, 33).

The reduction in feed intake was accompanied by decreased midgut and hindgut content, and elevated *FCR*, indicating reduced efficiency in converting ingested feed into biomass. This pattern was supported by reductions in biomass, condition factor, and specific growth rate (24), indicating broader physiological disturbances in the metabolic pathways regulating somatic growth and energy utilization. These outcomes are consistent with previous studies, which have shown that prolonged stress leads to a reallocation of energy resources away from growth and toward coping mechanisms, such as maintaining homeostasis and activating stress-related endocrine pathways (3, 4, 16, 22, 34).

In the present study, no significant differences in plasma cortisol levels (24) or hypothalamic *crf1* paralog expression were observed between control and chronically stressed fish (UCS) after 21 days, suggesting either HPI axis habituation or recovery. Despite this, a significant downregulation of the orexigenic neuropeptide *npya1* was detected in the UCS group, indicating a persistent suppression of appetite-promoting signals. Notably, we also observed a reduction in the anorexigenic transcript *cart2b* mRNA levels in the UCS fish, which may reflect a compensatory response to prolonged appetite suppression to save energy. Similar dual regulation patterns have been reported under feeding restriction in Atlantic salmon (35, 36).

### Acute stress effect

Acute stress exposure revealed altered gastrointestinal dynamics, particularly in the UCS group. Midgut content decreased more rapidly post-stress compared to controls, suggesting heightened gut motility. This is consistent with mammalian responses where stress increases gastric activity and peristalsis (37). Similarly, studies on Atlantic salmon have shown that acute stress—such as 15 min of confinement—can lead to substantial changes in the ultrastructure of enterocytes within the gastrointestinal tract, thereby affecting its functionality (38). In contrast, stress induced on Atlantic salmon by repeated samplings in the tanks significantly elevated plasma cortisol levels but did not significantly alter gastrointestinal transit rate (39). Subsequent statistical analysis of the present data employing Pearson’s correlation demonstrated a robust positive linear association between wet and dry content in both experimental groups. This consistency suggests that water intake was steady, and the noted drop in gastrointestinal content was mostly attributable to reduced feed consumption rather than changes in hydration.

The acute stressors also elicited a significant upregulation of the *crf1* paralogs in the UCS group, whereas no such changes were observed in the control group subjected to the same acute challenge. Notably, the expression of *crf1a1*, *crf1a2*, *crf1b1*, and *crf1b2* was time-dependent, with a marked increase observed after 60 min post-stress and peaking at 240 min. Similarly, *npya1* and *npya2* mRNA levels were upregulated in the UCS fish but not in control fish, suggesting the activation of a counter-regulatory mechanism aimed at mitigating chronic stress-induced anorexia. This supports our earlier suggestion of a compensatory role for *npy* in Atlantic salmon, as fish exposed to novel acute stress showed an increase of *npy* expression, which was also associated with an increase of *crf1b1* mRNA levels in the same experimental group (19). Similarly, increased *npy* mRNA levels were observed in the hypothalamus of rainbow trout and in the brain of zebrafish subjected to acute stress (17, 40, 41). No changes in the mRNA neuropeptides levels were observed in the control group, highlighting that the fish stress history may influence the basis for the regulation of the appetite-regulatory mechanisms under new upcoming stressful conditions. The current results align with earlier studies in teleosts, where *npy* has been consistently identified as a key orexigenic signal, particularly responsive to negative energy balance and stress-induced anorexia (42). Following acute stress exposure, *cart2b* levels in the UCS group increased, eventually reaching levels comparable to the control group from 60 min post-stress. This feedback regulation suggests a compensatory mechanism activated by acute stress, potentially aimed at restoring homeostatic balance in appetite-regulating pathways.

No changes in hypothalamic mRNA levels of *agrp1*, *pomca1*, and *pomca2* were observed. In contrast, previous research reported that exposure to acute stress increased *pomc1* and *agrp1* mRNA expression in the brain of zebrafish (17). In rainbow trout, however, acute stress decreased hypothalamic *pomc* expression, while *cart* remained unchanged (41).

### Additional considerations

The observed upregulation of *crf1* paralogs in this study appears to be associated with a concurrent temporal increase in plasma Acth levels in the same individuals (24), indicating a functional activation of the HPI axis. Traditionally, Crf neurons located in the POA are considered central to initiating this axis, leading to Acth release and subsequent cortisol production. However, it is important to consider that Crf-expressing neurons are also present in other hypothalamic regions, such as NLT (43), which is implicated in feeding regulation in Atlantic salmon (44). These Crf expressing neurons may contribute to Acth synthesis through mechanisms that are not strictly stress-related but instead linked to other metabolic or appetite-related cues. Intriguingly, despite the increase in *crf1* expression and Acth levels, plasma cortisol concentrations decreased at the same time points (24). This dissociation suggests the possibility that *crf* upregulation may not be driven by classical cortisol-mediated negative feedback. Supporting this, earlier studies in goldfish demonstrated that cortisol regulates *crf1* expression specifically within the POA (45), a region not included in the hypothalamic sections analyzed in the current study. Therefore, the observed *crf1* upregulation may reflect a localized hypothalamic response to acute stress or metabolic signals, independent of systemic cortisol feedback, highlighting the complexity and regional specificity of Crf signaling in teleost fish.

## Conclusions

This study showed that the interaction between the HPI axis and the appetite-regulating hypothalamic melanocortin system plays a key role in modulating feed intake during stress in Atlantic salmon. While appetite suppression emerged as the predominant response, the nature of this crosstalk varies depending on the duration of stress exposure, with distinct regulatory patterns observed under acute versus chronic stress conditions. Repeated exposure to stress during the chronic phase appeared to lead to desensitization or habituation of the HPI axis, as evidenced by the lack of significant changes in plasma cortisol levels and *crf1* mRNA paralog expression compared to the unstressed control group at the termination of the chronic stress period. Nevertheless, the continued suppression of feed intake and downregulation of the orexigenic *npya1* transcript mRNA levels indicate that central appetite-regulatory circuits remain affected. Additionally, the reduction in *cart2b* mRNA expression may reflect a compensatory attempt to restore feeding behavior, suggesting that the melanocortin system is actively responding to prolonged anorexia. In contrast, exposure to a novel acute stressor elicited a transient upregulation of all *crf1* mRNA paralogs, indicating that the fish remained responsive to new acute challenges despite prior habituation. Concurrent upregulation of the mRNA levels of orexigenic neuropeptides *npya1* and *npya2* suggests the activation of counter-regulatory mechanisms aimed at mitigating stress-induced anorexia, while the normalization of *cart2b* mRNA expression to control levels may reflect a compensatory response triggered by acute stress, potentially contributing to the restoration of homeostatic balance in appetite-regulating pathways. Overall, these findings reveal that the interaction between stress and appetite-regulatory systems in Atlantic salmon is dynamic and influenced by the duration of stress exposure, with acute and chronic stress eliciting distinct physiological responses suggesting adaptive regulatory mechanisms over time.

## Data Availability

The original contributions presented in the study are publicly available on the following link: https://dataverse.harvard.edu/dataset.xhtml?persistentId=doi:10.7910/DVN/VBYSHX.

## References

[1] Pankhurst NW Ludke SL King HR Peter RE. The relationship between acute stress, food intake, endocrine status and life history stage in juvenile farmed Atlantic salmon, Salmo salar. Aquaculture. (2008) 275:311–8. doi: 10.1016/j.aquaculture.2008.01.001

[2] Remen M Oppedal F Torgersen T Imsland AK Olsen RE. Effects of cyclic environmental hypoxia on physiology and feed intake of post-smolt Atlantic salmon: Initial responses and acclimation. Aquaculture. (2012) 326–329:148–55. doi: 10.1016/j.aquaculture.2011.11.036

[3] Madaro A Olsen RE Kristiansen TS Ebbesson LOE Nilsen TO Flik G. Stress in Atlantic salmon: response to unpredictable chronic stress. J Exp Biol. (2015) 218:2538–50. doi: 10.1242/jeb.120535, PMID: 26056242

[4] Lai F Budaev S Hundven IK Balseiro P Handeland SO Rønnestad I. Influence of water temperature on feed intake, appetite control, and energy allocation in Atlantic salmon (*Salmo salar*) post-smolt. Front. Physiol. (2025) 16:1–12. doi: 10.3389/fphys.2025.1646208, PMID: 40881654 PMC12380893

[5] Liland NS Lai F Sicuro A Azevedo ML Araujo P Hagen C. On-growing Atlantic salmon (*Salmo salar* L.) acclimate behaviourally and physiologically to chronic hypoxia, but exhibit reduced feed intake, growth and lipid retention. Aquaculture. (2026) 611. doi: 10.1016/j.aquaculture.2025.743043

[6] Grone BP Maruska KP. Divergent evolution of two corticotropin-releasing hormone (CRH) genes in teleost fishes. Front Neurosci. (2015) 9:365. doi: 10.3389/fnins.2015.00365, PMID: 26528116 PMC4602089

[7] Cardoso JCR Bergqvist CA Félix RC Larhammar D. Corticotropin-releasing hormone family evolution: Five ancestral genes remain in some lineages. J Mol Endocrinol. (2016) 57:73–86. doi: 10.1530/JME-16-0051, PMID: 27220618

[8] Faught E Aluru N Vijayan MM. The molecular stress response. In: Fish Physiology. Academic Press, London: Elsevier Inc (2016). 35:113–66. doi: 10.1016/B978-0-12-802728-8.00004-7

[9] Balasch JC Tort L. Netting the stress responses in fish. Front Endocrinol (Lausanne). (2019) 10:62. doi: 10.3389/fendo.2019.00062, PMID: 30809193 PMC6379254

[10] Wendelaar Bonga SE. The stress response in fish. (1997) 77:591–625. doi: 10.1152/physrev.1997.77.3.591, PMID: 9234959

[11] Hostetler CM Ryabinin AE. The crf system and social behavior: A review. Front Neurosci. (2013) 7:1–15. doi: 10.3389/fnins.2013.00092, PMID: 23754975 PMC3668170

[12] Rønnestad I Gomes AS Murashita K Angotzi R Jönsson E Volkoff H. Appetite-controlling endocrine systems in teleosts. Front Endocrinol (Lausanne). (2017) 8:73. doi: 10.3389/fendo.2017.00073, PMID: 28458653 PMC5394176

[13] Soengas JL Comesaña S Conde-Sieira M Blanco AM. Hypothalamic integration of nutrient sensing in fish. J Exp Biol. (2024) 227. doi: 10.1242/jeb.247410, PMID: 39082186

[14] Bernier NJ. The corticotropin-releasing factor system as a mediator of the appetite-suppressing effects of stress in fish. Gen Comp Endocrinol. (2006) 146:45–55. doi: 10.1016/j.ygcen.2005.11.016, PMID: 16410007

[15] Conde-Sieira M Chivite M Míguez JM Soengas JL. Stress effects on the mechanisms regulating appetite in teleost fish. Front Endocrinol (Lausanne). (2018) 9:631. doi: 10.3389/fendo.2018.00631, PMID: 30405535 PMC6205965

[16] Bernier NJ Bedard N Peter RE. Effects of cortisol on food intake, growth, and forebrain neuropeptide Y and corticotropin-releasing factor gene expression in goldfish. Gen Comp Endocrinol. (2004) 135:230–40. doi: 10.1016/j.ygcen.2003.09.016, PMID: 14697310

[17] Cortés R Teles M Oliveira M Fierro-Castro C Tort L Cerdá-Reverter JM. Effects of acute handling stress on short-term central expression of orexigenic/anorexigenic genes in zebrafish. Fish Physiol Biochem. (2018) 44:257–72. doi: 10.1007/s10695-017-0431-7, PMID: 29071448

[18] Doyon C Gilmour KM Trudeau VL Moon TW. Corticotropin-releasing factor and neuropeptide Y mRNA levels are elevated in the preoptic area of socially subordinate rainbow trout. Gen Comp Endocrinol. (2003) 133:260–71. doi: 10.1016/S0016-6480(03)00195-3, PMID: 12928015

[19] Lai F Comesaña S Gomes AS Flatejord D Tolås I Espe M. Anorectic role of high dietary leucine in farmed Atlantic salmon (*Salmo salar* L.): Effects on feed intake, growth, amino acid transporters and appetite-control neuropeptides. Aquaculture. (2023) 566. doi: 10.1016/j.aquaculture.2022.739204

[20] Kalananthan T Lai F Gomes AS Murashita K Handeland S Rønnestad I. The melanocortin system in atlantic salmon (*Salmo salar* L.) and its role in appetite control. Front Neuroanat. (2020) 14:48. doi: 10.3389/fnana.2020.00048, PMID: 32973463 PMC7471746

[21] Kalananthan T Gomes AS Lai F Tolås I Jordal AEO Norland S. Brain Distribution of 10 cart Transcripts and Their Response to 4 Days of Fasting in Atlantic Salmon (*Salmo salar* L.). Front Mar Sci. (2021) 8:763766. doi: 10.3389/fmars.2021.763766

[22] Lai F Royan MR Gomes AS Espe M Aksnes A Norberg B. The stress response in Atlantic salmon (*Salmo salar* L.): identification and functional characterization of the corticotropin-releasing factor (crf) paralogs. Gen Comp Endocrinol. (2021) 313. doi: 10.1016/j.ygcen.2021.113894, PMID: 34478716

[23] Tolås I Kalananthan T Gomes AS Lai F Norland S Murashita K. Regional Expression of npy mRNA Paralogs in the Brain of Atlantic Salmon (*Salmo salar* L.) and Response to Fasting. Front Physiol. (2021) 12:720639. doi: 10.3389/fphys.2021.720639, PMID: 34512390 PMC8427667

[24] Madaro A Lai F Gunnar P Hansen T Gelebart V Muren B. Comparing physiological responses of acute and chronically stressed diploid and triploid Atlantic salmon (*Salmo salar)* . Aquaculture Reports (2024) 36. doi: 10.1016/j.aqrep.2024.102041

[25] Del Vecchio G Lai F Gomes AS Verri T Kalananthan T Barca A. Effects of Short-Term Fasting on mRNA Expression of Ghrelin and the Peptide Transporters PepT1 and 2 in Atlantic Salmon (*Salmo salar*). Front Physiol. (2021) 12:666670. doi: 10.3389/fphys.2021.666670, PMID: 34234687 PMC8255630

[26] Helland SJ Grisdale-Helland B Nerland S. A simple method for the measurement of daily feed intake of groups of fish in tanks. Aquaculture. (1996) 139:157–63. doi: 10.1016/0044-8486(95)01145-5

[27] Kalananthan T Murashita K Rønnestad I Ishigaki M Takahashi K Silva MS. Hypothalamic agrp and pomc mRNA Responses to Gastrointestinal Fullness and Fasting in Atlantic Salmon (*Salmo salar*, L.). Front Physiol. (2020) 11:61. doi: 10.3389/fphys.2020.00061, PMID: 32116771 PMC7026680

[28] McEwen BS Stellar E. Stress and the individual: mechanisms leading to disease. Arch Intern Med. (1993) 153:2093–101. doi: 10.1001/archinte.1993.00410180039004 8379800

[29] Mc Ewen BS. Stress, adaptation, and disease: allostasis and allostatic load. Ann N Y Acad Sci. (1998) 840:33–44. doi: 10.1111/j.1749-6632.1998.tb09546.x, PMID: 9629234

[30] Höglund E Sørensen C Bakke MJ Nilsson GE Overli O. Attenuation of stress-induced anorexia in brown trout (*Salmo trutta*) by pre-treatment with dietary l-tryptophan. Br J Nutr. (2007) 97:786–9. doi: 10.1017/S0007114507450280, PMID: 17349093

[31] Pickering AD Pottinger TG Christie P. Recovery of the brown trout, *Salmo trutta* L., from acute handling stress: a time-course study. J Fish Biol. (1982) 20:229–44. doi: 10.1111/j.1095-8649.1982.tb03923.x

[32] McCormick SD Shrimpton JM Carey JB O’Dea MF Sloan KE Moriyama S. Repeated acute stress reduces growth rate of Atlantic salmon parr and alters plasma levels of growth hormone, insulin-like growth factor I and cortisol. Aquaculture. (1998) 168:221–35. doi: 10.1016/S0044-8486(98)00351-2

[33] Vikeså V Nankervis L Hevrøy EM. Appetite, metabolism and growth regulation in Atlantic salmon (*Salmo salar* L.) exposed to hypoxia at elevated seawater temperature. Aquac Res. (2017) 48:4086–101. doi: 10.1111/are.13229

[34] Vindas MA Madaro A Fraser TWK Höglund E Olsen RE Kristiansen TS. Uncontrollable chronic stress reduces growth disparities in farmed Atlantic salmon. Physiol Behav. (2017) 179:246–52. doi: 10.1016/j.physbeh.2017.06.012, PMID: 28668622

[35] Kalananthan T Folkedal O Gomes AS Lai F Handeland SO Tolås I. Impact of long-term fasting on the stomach-hypothalamus appetite regulating genes in Atlantic salmon postsmolts. Aquaculture. (2023) 563. doi: 10.1016/j.aquaculture.2022.738917

[36] Lai F Rønnestad I Olsen TS Gelebart V Balseiro P Vågseth T. Adaptations to intermittent fasting in large sea caged Atlantic salmon (*Salmo salar*); effects on feeding, energy homeostasis, and growth. Aquaculture. (2025) 599. doi: 10.1016/j.aquaculture.2025.742181

[37] Stengel A Tache’ Y. Neuroendocrine control of the gut during stress: corticotropin-releasing factor signaling pathways in the spotlight. Annu Rev Physiol. (2009) 71:219–39. doi: 10.1146/annurev.physiol.010908.163221, PMID: 18928406 PMC2714186

[38] Olsen RE Sundell K Hansen T Hemre GI Myklebust R Mayhew TM. Acute stress alters the intestinal lining of Atlantic salmon, Salmo salar L.: An electron microscopical study. Fish Physiol Biochem. (2002) 26:211–21. doi: 10.1023/A:1026217719534

[39] Miles PC Mock TS Jago MK Salini MJ Smullen RP Francis DS. Validation of gut transit rate assessment methodology and the mitigation of sampling stress in Atlantic salmon, *Salmo salar* . Aquaculture. (2025) 596. doi: 10.1016/j.aquaculture.2024.741771

[40] Conde-Sieira M Agulleiro MJ Aguilar AJ Míguez JM Cerdá-Reverter JM Soengas JL. Effect of different glycaemic conditions on gene expression of neuropeptides involved in control of food intake in rainbow trout; Interaction with stress. J Exp Biol. (2010) 213:3858–65. doi: 10.1242/jeb.048439, PMID: 21037065

[41] Naderi F Hernández-Pérez J Chivite M Soengas JL Míguez JM López-Patiño MA. Involvement of cortisol and sirtuin1 during the response to stress of hypothalamic circadian system and food intake-related peptides in rainbow trout, *Oncorhynchus mykiss* . Chronobiol Int. (2018) 35:1122–41. doi: 10.1080/07420528.2018.1461110, PMID: 29737878

[42] Volkoff H. The neuroendocrine regulation of food intake in fish: A review of current knowledge. Front Neurosci. (2016) 10:540. doi: 10.3389/fnins.2016.00540, PMID: 27965528 PMC5126056

[43] Coto-Montes A Garcia-Fernandez DJM Del Brio MA Riera P. The distribution of corticotropin-releasing factor immunoreactive neurons and nerve fibres in the brain of *Gambusia affinis* and *Salmo trutta* . Histol Histopathol. (1994) 9:233–41., PMID: 8075480

[44] Norland S Eilertsen M Rønnestad I Helvik JV Gomes AS. Mapping key neuropeptides involved in the melanocortin system in Atlantic salmon (*Salmo salar*) brain. J Comp Neurol. (2023) 531:89–115. doi: 10.1002/cne.25415, PMID: 36217593 PMC9828751

[45] Bernier NJ Peter RE. Appetite-suppressing effects of urotensin I and corticotropin-releasing hormone in goldfish (Carassius auratus). Neuroendocrinology. (2001) 73:248–60. doi: 10.1159/000054642, PMID: 11340339

