# Supplementary material for: Cross-regulatory interaction between the HPI axis and appetite regulation in Atlantic salmon (Salmo salar) parr under chronic and acute stress
Source: Front Endocrinol (Lausanne). 2025 Nov 5;16:1685870. doi: 10.3389/fendo.2025.1685870 (PMC12626856; doi:10.3389/fendo.2025.1685870)
Supplement: Supplementary file 1 [file DataSheet1.docx]

Supplementary Figures and Tables to

**Cross-regulatory interactions between the HPI axis and appetite regulatory mechanisms in Atlantic salmon (*Salmo salar*) parr under chronic and acute stress conditions**

***Floriana Lai, Ivar Rønnestad, Lars Stien Helge, Angelico Madaro***

**Supplementary Table 1. Primer’s sequence used for qPCR mRNA expression in Atlantic salmon.** Amplicon sizes, qPCR efficiency and R2 are listed for each primer pair. F=forward; R=reverse.

**
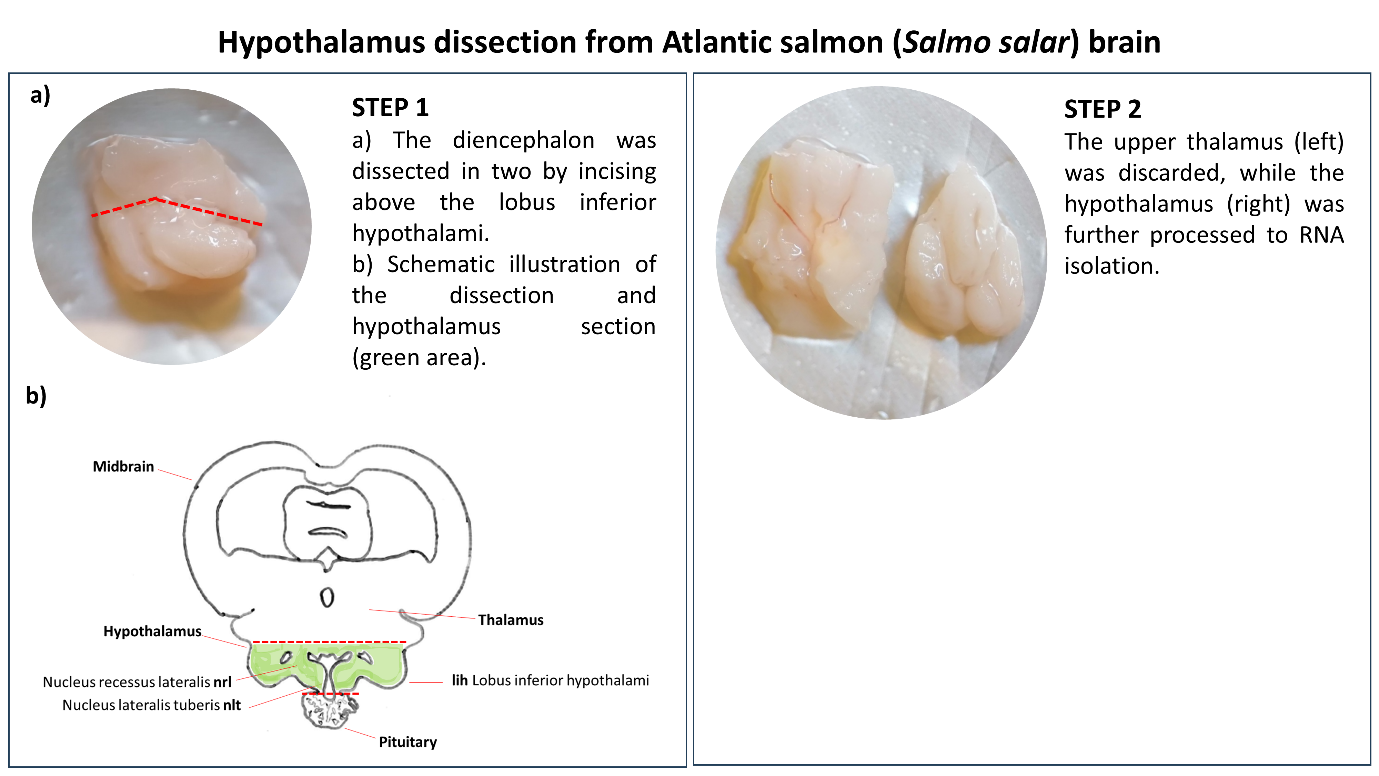
**

**Supplementary Figure 1**. **Atlantic salmon brain hypothalamus dissection.**

**Supplementary Figure 2.** **Daily feed intake of Atlantic salmon parr under chronic stress**. Fish were subjected to unpredictable chronic stress (UCS) for 21 days or left undisturbed (Control). n=3/day/group. Data represent mean ± SEM.

**Supplementary Figure 3**. **Total feed intake and feed conversion ratio (FCR) of Atlantic salmon parr after exposure to chronic stress.** Fish were exposed to unpredictable chronic stress (UCS) for 21 days or left undisturbed (Control). n=3/group. Data represent mean ± SEM.

**Supplementary Figure 4.** **Wet and dry gastrointestinal fullness of Atlantic salmon parr**. Fish were subjected to unpredictable chronic stress (UCS) for 21 days or left undisturbed (Control). n=50/group for the wet content. n=49/control and n=48/UCS for the dry content. Data represent mean ± SEM.

**Supplementary Figure 5**. **Pearson r correlation between the wet and dry content in the stomach, midgut and hindgut of Atlantic salmon parr.** Fish were exposed to unpredictable chronic stress (UCS) for 21 days or left undisturbed (Control). n=3/group. n=49 for the control, n=48 for the UCS group. Data represent mean ± SEM.

**Supplementary Figure 6. Hypothalamic *crf1* mRNA levels of Atlantic salmon parr after exposure to a novel acute stressor**. Fish were subjected to unpredictable chronic stress (UCS) for 21 days or left undisturbed (Control). n=9-10/time point in the control group; n=8-9/time point in the UCS group.* indicates significant differences between the control and UCS group at the same time point. a-b letters indicate significant differences from time 0 within the same experimental group.


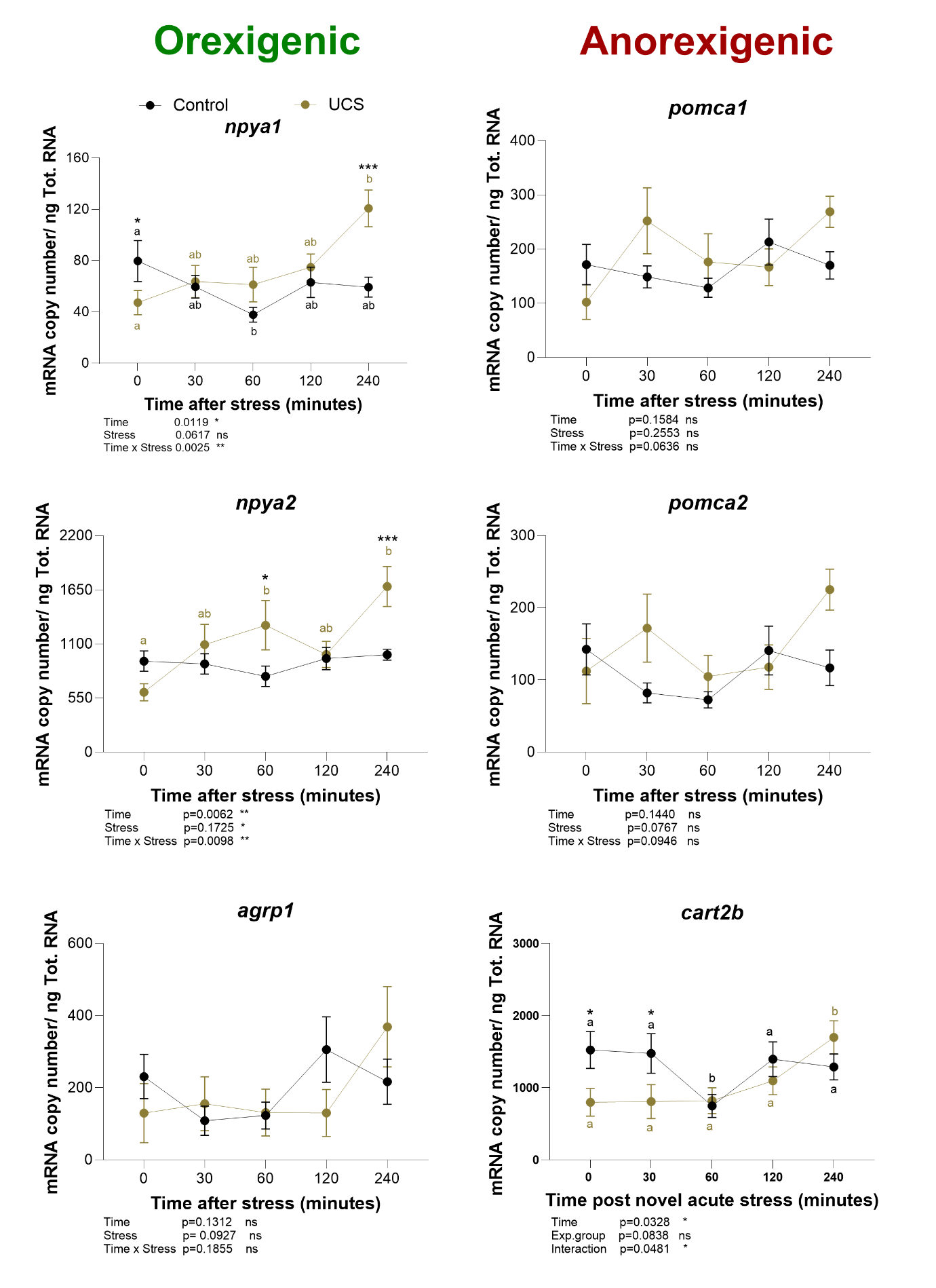


**Supplementary Figure 7. Hypothalamic appetite regulatory genes in Atlantic salmon parr after exposure to a novel acute stressor**. Fish were subjected to unpredictable chronic stress (UCS) for 21 days or left undisturbed (Control). n=9-10/time point in the control group; n=8-9/time point in the UCS group. * indicates significant differences between the control and UCS group at the same time point. a-b letters indicate significant differences from time 0 within the same experimental group.

**Supplementary Figure 8**. **Dry gastrointestinal content of Atlantic salmon parr exposed to a novel acute stress**. Fish were exposed to unpredictable chronic stress (UCS) for 21 days or left undisturbed (Control). n=3/group. n=10 group/time point. Data represent mean ± SEM. * indicates significant differences between the control and UCS group at the same time point. a-b letters indicate significant differences from time 0 within the same experimental group.
